# Difference between Microscopic and PCR Examination Result for Malaria Diagnosis and Treatment Evaluation in Sumba Barat Daya, Indonesia

**DOI:** 10.3390/tropicalmed7080153

**Published:** 2022-07-29

**Authors:** Dwita Anastasia Deo, Elizabeth Henny Herningtyas, Umi Solekhah Intansari, Taufik Mulya Perdana, Elsa Herdiana Murhandarwati, Marsetyawan H. N. E. Soesatyo

**Affiliations:** 1 Doctoral Program in Medicine and Health, Faculty of Medicine, Public Health and Nursing, Universitas Gadjah Mada, Yogyakarta 55281, Indonesia; dwita_2011@mail.ugm.ac.id; 2 Departement of Parasitology, Faculty of Medicine, Universitas Nusa Cendana, Kupang 85001, Indonesia; 3 Department of Clinical Pathology and Laboratory Medicine, Faculty of Medicine, Public Health and Nursing, Universitas Gadjah Mada, Yogyakarta 55281, Indonesia; ehennyh@ugm.ac.id (E.H.H.); intansariumi@gmail.com (U.S.I.); 4 Departement of Parasitology, Faculty of Medicine, Public Health and Nursing, Universitas Gadjah Mada, Yogyakarta 55281, Indonesia; taufik.mulya.p@mail.ugm.ac.id; 5 Center for Tropical Medicine, Faculty of Medicine, Public Health and Nursing, Universitas Gadjah Mada, Yogyakarta 55281, Indonesia; 6 Department of Histology, Faculty of Medicine, Public Health and Nursing, Universitas Gadjah Mada, Yogyakarta 55281, Indonesia; marshnes@ugm.ac.id

**Keywords:** subclinical malaria, asymptomatic malaria, high endemicity, nested PCR, microscopic examination

## Abstract

Microscopic examination is the backbone of malaria diagnosis and treatment evaluation in Indonesia. This test has limited ability to detect malaria at low parasite density. Inversely, nested polymerase chain reaction (PCR) can detect parasites at a density below the microscopic examination’s detection limit. The objective of this study is to compare microscopic and PCR results when being used to identify malaria in suspected patients and patients who underwent dihydroartemisinin–piperaquine (DHP) therapy in the last 3–8 weeks with or without symptoms in Sumba Barat Daya, Nusa Tenggara Timur, Indonesia. Recruitment was conducted between April 2019 and February 2020. Blood samples were then taken for microscopic and PCR examinations. Participants (*n* = 409) were divided into three groups: suspected malaria (42.5%), post-DHP therapy with fever (4.9%), and post-DHP therapy without fever (52.6%). Microscopic examination found five cases of *P. falciparum* + *P. vivax* infection, while PCR found 346 cases. All microscopic examinations turned negative in the post-DHP-therapy group. Conversely, PCR result from the same group yielded 29 negative results. Overall, our study showed that microscopic examination and PCR generated different results in detecting *Plasmodium* species, especially in patients with mixed infection and in patients who recently underwent DHP therapy.

## 1. Introduction

In recent years, malaria cases in Indonesia have been showing a declining trend. In fact, more than half of the districts in this country were free from malaria in 2017 [1]. This is a major milestone for the Indonesian malaria elimination campaign that aims to free the country from malaria in 2030. However, the success of the malaria elimination campaign was not distributed evenly. For instance, Nusa Tenggara Timur (NTT), the province with the second highest number of malaria cases in 2020, recorded 15,000 malaria cases [2]. While the Annual Parasite Incidence (API) is decreasing in NTT from 14.82‰ in 2014 to 2.88‰ in 2020, several districts recorded much higher API [3]. For instance, a district in this province named Sumba Barat Daya showed an API of 20.92‰ in 2020 [3]. Challenges in diagnosis, case management, and surveillance, along with vector control, are thought to hinder elimination efforts in these districts. 

As a primary health center (PHC) in Sumba Barat Daya District, Kori PHC reported 1,343 cases of malaria in 2020, rising sharply from 487 cases in 2019 [3,4]. Furthermore, the same reports also mentioned that in the two years [3,4], the Slide Positivity Rate and Annual Blood Examination Rate in the working area of this PHC are far from the government standard. As such, we suspected that the number of malaria cases in the Kori PHC working area might be underestimated. This suspicion was supported by field observation that found that several residents, especially those who live near forest borders, rivers, and gardens, may experience symptoms associated with malaria—such as myalgia, cephalgia, and fever—several times a year. However, in many of these patients, no parasite was found upon microscopic examination. Hence, a question emerged as to whether these residents suffered from submicroscopic parasitemia.

Indonesian national guideline recommends dihydroartemisinine–piperaquine (DHP; each tablet contains 40 mg dihydroartemisinine +320 mg piperaquine phosphate) fixed-dose combination with or without 0.25 mg/kg body weight of primaquine to treat uncomplicated malaria [5]. DHP is a safe and effective treatment for acute uncomplicated malaria [6,7,8]. However, DHP is ineffective in combating the gametocyte and hypnozoite stage of parasites [6,9]. As such, primaquine was added to the treatment regimen to target the two parasite stages. In Indonesia, a single dose of primaquine was added to a 3-day-course of DHP when a patient was infected by *P. falciparum* alone [5]. For patients who suffered from *P. malariae* mono-infection, DHP alone for 3 days was given [5]. As for patients who suffered from *P. vivax* or *P. ovale* infection, whether it was mono or mixed infection, a 14-day course of primaquine was given in addition to a 3-day course of DHP [5]. Administration of treatment must be directly observed by a family member who lives under the same roof. The family member must then report the drug administration to the local/village malaria cadre, who in turn will report to the PHC. Given how the treatment is species-specific, false negative due to low parasite density might lead to inappropriate treatment and, ultimately, persistence.

Microscopic examination is the backbone of malaria diagnosis in Indonesian PHCs. This method can distinguish parasite species and stages, quantify parasite density, and is inexpensive [10]. In endemic areas, malaria diagnosis using microscopic examination to a density of 200 parasites/µL blood can reliably diagnose clinically important cases [11]. Detecting Plasmodium sp at density of <50 parasites/µL blood might only be achieved by experienced staff [12]. Unlike microscopic examination, PCR examination is able to detect parasites down to <5 parasites/µL blood [13]. The better sensitivity of PCR, especially in cases with low parasite density or mixed infection [14,15,16,17,18], may reduce the error in malaria diagnosis when used appropriately.

This cross-sectional study aimed to compare the results from microscopy and PCR examination when being used to detect *Plasmodium* sp. among the residents of Kodi Utara Subdistrict, Sumba Barat Daya District. By doing this, we were able to identify cases of asymptomatic malaria in this population and identify the Plasmodium species infecting these patients.

## 2. Materials and Methods

### 2.1. Study Design

This study utilized a cross-sectional design. Participants were divided into 3 groups: suspected malaria, post-DHP therapy with fever, and post-DHP therapy without fever. For each participant, blood samples were taken for microscopic and PCR examination. The results of both diagnostic modalities were then compared to each other. 

### 2.2. Study Subjects and Sample Collection

The population of this study is the resident of Kodi Utara Subdistrict, Sumba barat Daya District at Sumba Island, NTT. Participants were recruited from patients that visited Kori PHC as well as residents who recently underwent DHP therapy that were followed up by a local malaria cadre. Recruitment was conducted between April 2019 and February 2020. The inclusion criteria for this study are local residents of Kodi Utara Subdistricts who were either suspected of malaria or just recently underwent DHP therapy with or without primaquine in the last 3–8 weeks. In addition, we excluded patients who were suspected of suffering from severe malaria, pregnant women, and resident who declined to participate. Participants (*n* = 409) who fulfilled inclusion criteria were divided into three groups: those who were suspected of malaria (42.5%; *n* = 174), post DHP therapy with fever (4.9%; *n* = 20), and post-DHP therapy without fever (52.6%; *n* = 215).

Characteristics of the participants were taken using a questionnaire that asked about the participant’s age, gender, history of malaria, history of DHP therapy within the last 2 years, and their living area. Participants were then physically examined and had their venous and peripheral blood samples drawn. Venous blood samples were drawn into EDTA tubes for molecular examination. Peripheral blood samples were taken from the participant’s fingertip and were directly made into slides for microscopic examination. Examination of thick and thin blood smears with a microscope was carried out at the Kori PHC laboratory, while the molecular examination was performed in the Parasitology Laboratory, Faculty of Medicine, Public Health, and Nursing, Universitas Gadjah Mada (FMPHN-UGM), Yogyakarta, Indonesia.

### 2.3. Microscopic Method

Thin and thick slides of peripheral blood were made after collection and allowed to air dry. Slides were stained with 3% Giemsa solution for 45 min at room temperature [19]. Slides were then examined using a compound light microscope under ×100 objective lens (oil-immersion) magnification and 10× ocular lens by two independent certified microscopists (level 1) in the Kori PHC and Sumba Barat Daya District Health Office [20]. All slides were examined for a minimum of 100 high-magnification fields before being recorded as negative, low density, mono, and mixed-species infections. 

### 2.4. Molecular Method

The DNA was extracted from venous blood collected in an EDTA tube using a commercial kit (Geneaid Kit) and stored at −20 °C. Using the 18s ribosomal RNA [21] as a reference, gene-based nested PCR was performed with primers and cycling conditions as described for nested PCR. The species-specific nucleotide sequences of the *P. falciparum*, *P. vivax*, *P. ovale,* and *P. malariae* were amplified as described previously with slight modifications [22]. The volume of the PCR reaction was 30 µL containing 15 µL Tag green mix, 1 µL each primer, 10 µL dH2O, and 3 µL DNA template. The result of nested PCR with two amplifications and species separated by electrophoresis on 2% agarose gel, 0.5× TBE dilution, 100 volts, for 30 min with 5 µL FluoroVue (Smobio, Taiwan, China) staining and ultraviolet transillumination was used for band visualization. DNA extraction and nested PCR examination were conducted at the Parasitology Laboratory, FMPHN-UGM, Yogyakarta, Indonesia.

### 2.5. Statistical Analysis

The results of microscopic examination and PCR were analyzed by McNemar’s test to assess whether the proportions differed from repeated measurements in one sample.

### 2.6. Definitions Used in the Study

Asymptomatic malaria was defined as an asymptomatic individual whose microscopic and/or molecular examination results show the presence of *Plasmodium* sp. [23,24]. Microscopic parasitemia was defined as a positive test result by microscopic examination as well PCR. Submicroscopic parasitemia was defined as a negative test result by microscopic examination but a positive test result by PCR. Suspected malaria was defined as an individual who was suspected by a physician to suffer from malaria, generally due to the presence of body temperature > 37.5 °C with or without other symptoms [24]. Post-DHP therapy with fever was an individual who had finished taking DHP and had a body temperature >37.5 °C.

## 3. Results

Participants (*n* = 409) were divided into three groups: suspected of malaria (42.5%; *n* = 174), post-DHP therapy with fever (4.9%; *n* = 20), and post-DHP therapy without fever (52.6%; *n* = 215). Characteristics of these participants are shown in Table 1.

Most participants in suspected malaria and post-DHP therapy with fever group are aged 5–15. In the post-DHP therapy without fever group, most participants are above 15 years of age. In all groups, most participants are male and had multiple histories of malaria. Only 10 participants had no previous history of DHP therapy. Participants belonging to the suspected malaria and post DHP therapy without fever mostly live near forest and garden borders. Meanwhile, most participants in the post-DHP therapy with fever group live near tributaries.

Plasmodium species identification from the microscopic examination were compared to molecular examination across the three participant groups. The result is presented in Table 2.

Plasmodium species were not found by microscopic examination in patients that underwent DHP therapy, regardless of the presence of fever. However, PCR examination showed different results. Samples from post-DHP therapy patients with fever showed submicroscopic parasitemia that contained *P. falciparum* (0.7%; *n* = 3), *P. vivax* (3.2%; *n* = 1), and *P. falciparum + P. vivax* (3.9%; *n* = 16). Meanwhile, samples from post-DHP therapy patients without fever showed the presence of *P. vivax* (0.5%; *n* = 2) and *P. falciparum + P. vivax* (44.9%; *n* = 184), even though the patients were asymptomatic during the presentation. *P. ovale* were not identified in any sample by both microscopic and PCR examination. Finally, only 29 (7.1%) samples were found to contain no parasite by both microscopic and PCR examination.

The microscopic examination result from the suspected malaria group suggested that mono infection by *P. falciparum* was the leading cause of illness (73.6%; *n* = 128). However, PCR results showed that mixed infection by *P. falciparum + P. vivax* was instead the leading cause of illness in this group (83.9%; *n*= 146). Meanwhile, microscopic examination seemed to miss six cases of mixed *P. falciparum + P. vivax + P. malariae* detected by PCR. Instead, these cases were identified as mono infection by *P. malariae*.

McNemar test showed a significant difference between the result of microscopic examination and nested PCR. Overall, microscopic examination found 128 *P. falciparum* mono infections, while nested PCR only found 11 (*p* < 0.001). *P. vivax* mono infection was found in 34 samples by microscopic examination and in 16 samples by nested PCR (*p* < 0.001). Microscopic examination found mixed *P. falciparum + P. vivax* infection in five samples, but nested PCR results showed 346 samples were infected with both species (*p* < 0.001). The results of the microscopic examination showed mono infection by *P. malariae* in seven samples, but nested PCR showed only one sample was infected *P. malariae* alone (*p* = 0.031). Six cases of mixed infection by *P. falciparum + P. vivax + P. malariae* were found by PCR. However, the microscopic examination did not find any samples infected by these groups of pathogens. Most microscopic examinations in this study yielded negative results (*n* = 235). However, only 29 samples examined by nested PCR returned negative results (*p* < 0.001).

Comparison between the results of microscopic examination and nested PCR when participants visited the Kori PHC are shown in Table 3.

## 4. Discussion

The result of our study showed that microscopic examination and PCR have visibly different results when being used to detect parasitemia. This is especially true in cases of mixed infection and in groups of patients who recently underwent DHP therapy.

In our study, microscopic examination was only able to identify five mixed infections, while PCR found 351 mixed infections. The limitation of microscopic examination in detecting mixed infection has been well-documented [15,17,18]. A recent meta-analysis estimated that the overall sensitivity and specificity of microscopic examination against PCR when being used to diagnose malaria is 75.20% and 97.12%, respectively [16]. However, it appears that microscopic examination showed lower diagnostic accuracy when being used to assess malaria in asymptomatic patients and in cases of mixed infection. For instance, Golassa et al. [14] estimated that when being used to detect asymptomatic malaria, microscopic examination has a sensitivity of 16.5% and a specificity of 24.2% compared to PCR. Meanwhile, Ehtesham et al. [17] found that against PCR, the sensitivity of microscopic examination to detect mixed infection was only 16.6%. The diagnostic accuracy of microscopic examination itself heavily relies on the skill of the examiner, quality of reagent, quality of microscope, parasite density, and quality control system [12,25]. As such, several efforts have been proposed to improve the diagnostic performance of microscopic examination. Odhiambo et al., for example, suggested that systematically refreshing the training of microscopist significantly improves the diagnostic accuracy of microscopic examination [26]. Another suggested improvement is the utilization of saponin hemolysis to artificially increase the parasite density. This method allows microscopy to perform as well as PCR in diagnosing mixed malaria infection [27]. However, there is a lack of evidence regarding its utility under field conditions, and thus, further studies are required.

The dominant species found in our study is *P. vivax*, with the majority of them occurring in the form of mixed *P. falciparum + P. vivax* infection. This is in contrast to the local government report, which mentioned that 68% of malaria cases in NTT province—where this study was conducted—was caused by *P. falciparum*, with *P. vivax* contributing to only 26% of the case [3]. However, it should be noted that this report was built up primarily using data collected through microscopic examination. Indeed, assessing the true extent of *P. vivax* distribution is difficult, especially using microscopic examination. This is because *P. vivax* infection tends to be asymptomatic and has low parasite density, which may lead to false negative microscopic examination result [28].

Owing to its ability to detect parasites at lower parasite density than microscopic examination, molecular methods such as PCR can be used as an alternative epidemiological surveillance method. Several Indonesian studies have employed this strategy, with varying results. For example, surveillance conducted in North Sumatra province in 2015 revealed that *P. vivax* (33.9%) is the most dominant species in this region, with *P. falciparum* found in only 24.8% of cases [29]. A similar survey conducted on Flores Island, NTT, in 2008 revealed that mono-infection by *P. falciparum* and *P. vivax* was found in 43.1% and 39.6% of positive samples, respectively [30]. A smaller study conducted in the Anak Dalam Tribe in Jambi Province found *P. vivax* mono-infection in 33 out of 35 positive samples [31]. Unfortunately, similar studies from other parts of Indonesia are still limited. Due to the dynamic nature of the disease and improvement of malaria control measures, investigating *Plasmodium* species epidemiology in Indonesia using molecular methods is a path worth exploring.

Due to its ability to form dormant hypnozoites, management of *P. vivax* at community level is challenging. In fact, Adekulne, et al. estimated that more than 70% of *P. vivax* infections in Thailand and Papua New Guinea arise from hypnozoite reactivation [32]. The relapse pattern found in Indonesia is believed to be caused by the Chesson strain [33]. This strain is known to produce a frequent relapse pattern, with the majority of volunteers infected by this strain relapsed 30 days following primary attack [34]. 

The frequency of relapse can be suppressed by administering anti-malaria that targets hypnozoite stage of *P. vivax*. The combination of DHP with or without primaquine is the mainstay of therapy for uncomplicated malaria in Indonesia [5]. For *P. falciparum* infection, primaquine was given as a single dose, while a 14-day daily dose of primaquine was given for *P. vivax* infection [5]. In our study, most *P. vivax* infections were missed by microscopic examination. Consequently, these patients did not receive proper primaquine dosing. Given that DHP could not eliminate hypnozoite [6], we strongly suspect that the recurrence of malaria-associated symptoms among residents in our study site might stem from improperly treated hypnozoite of *P. vivax*. This suspicion is supported by our findings in the post-DHP therapy group.

In the post-DHP therapy group, most patients received positive PCR results for mixed *P. falciparum + P. vivax* infection despite negative microscopic examination results. This suggests that recent administration of DHP likely reduced—but did not eliminate—Plasmodium sp. in patient’s blood to below the detection threshold of microscopy. Regardless, delayed conversion of PCR results following anti-malarial therapy was also observed in other studies. For instance, Vafa Homann et al. [35] found positive microscopic examination and quantitative polymerase chain reaction (qPCR) result for up to 2 and 48 days, respectively, following artemether–lumefantrine treatment. Another study found that the mean duration of parasitemia as measured by microscopic examination and qPCR was 2.2 and 7.9 days, respectively [36].

Interpretation of PCR results following the administration of therapy must be made cautiously, as detection of genetic material from the dead parasite is possible. A mice study suggested that parasite DNA was rapidly cleared from the mice’s circulation following parasite killing [28]. However, a Swedish study estimated that up to 48 days were needed to clear *P. falciparum* DNA from a patient’s circulation following initiation of therapy [35]. Another study conducted in Tanzania found that in the absence of reinfection or recrudescence, the qPCR result for *P. falciparum* remains positive for at least 42 days following treatment initiation [37]. The study, however, did not explore the reason for this positive result [37]. Due to high number of *P. vivax* infections missed by microscopy, we suspect that it is unlikely that positive PCR results for this species in the post-DHP therapy group came from the remnant of the dead parasite.

Regardless, we acknowledge that this study has several limitations that may limit its interpretation. For instance, due to its cross-sectional design, interpreting the temporality in the relationship between PCR and microscopic examination results is not feasible. Furthermore, we could not quantify the parasite DNA recovered from our sample due to the type of PCR that we used. Taken together, these limitations prevent us from confirming the reason behind the discrepancy between PCR and microscopy results in our study.

We believe that it is important to verify whether the positive PCR in the post-DHP therapy group truly stem from the detection of *P. vivax* infection that was undiagnosed by microscopic examination. This can be done by utilizing the qPCR technique to see whether there is a fall in the amount of parasite DNA over time following the initiation of proper treatment. If undiagnosed *P. vivax* is truly the cause, then there is an urgent need to reform the detection method in the community to eliminate malaria from the region.

## 5. Conclusions

Microscopic examination and nested PCR have noticeably different results when being used to detect Plasmodium species. The difference in the result yielded by the two diagnostic modalities is especially apparent in cases of mixed infection and in groups of patients who recently underwent DHP therapy. Considering the limitation of microscopic examination and the outcome of our study, we believe that an evaluation of the malaria testing policy is needed.

## Figures and Tables

**Table 1:** Characteristics of participants with suspected malaria, post-DHP therapy with fever, and post-DHP therapy without fever.

Characteristics	Suspected Malaria (*n* = 174)	Post-DHP Therapy with Fever (*n* = 20)	Post-DHP Therapy without Fever (*n* = 215)
**Age**			
≤5 years old	15	1	3
5–15 years old	85	15	89
>15 years old	74	4	123
**Gender**			
Male	123	14	170
Female	51	6	45
**History of malaria**			
None	5	0	0
Once	47	7	50
More than once	122	13	165
**History of DHP ^1^ therapy** **in the past 2 years**			
Yes	164	20	215
No	10	0	0
**Living area**			
Forest and garden border	174	20	215
Tributary border	35	12	35

^1^ DHP—dihydroartemisinin–piperaquine.

**Table 2:** Plasmodium species identification results through microscopic and nested PCR of participants with suspected malaria, post-DHP therapy with fever and without fever.

Respondent Group	Suspected Malaria (*n* = 174)			Post-DHP Therapy with Fever (*n* = 20)			Post-DHP Therapy without Fever (*n* = 215)		
Classification Age (years old)	<5	5–15	>15	<5	5–15	>15	<5	5–15	>15
**Microscopy results**									
*P. falciparum*	13 (7.5%)	73 (42%)	42 (24.1%)	0 (0%)	0 (0%)	0 (0%)	0 (0%)	0 (0%)	0 (0%)
*P. vivax*	2 (1.2%)	10 (5.7%)	22 (12.6%)	0 (0%)	0 (0%)	0 (0%)	0 (0%)	0 (0%)	0 (0%)
*P. malariae*	0 (0%)	1 (0.6%)	6 (3.4%)	0 (0%)	0 (0%)	0 (0%)	0 (0%)	0 (0%)	0 (0%)
*P. falciparum + P. vivax*	0 (0%)	1 (0.6%)	4 (2.3%)	0 (0%)	0 (0%)	0 (0%)	0 (0%)	0 (0%)	0 (0%)
*P. falciparum + P. vivax + P. malariae*	0 (0%)	0 (0%)	0 (0%)	0 (0%)	0 (0%)	0 (0%)	0 (0%)	0 (0%)	0 (0%)
Negative	0 (0%)	0 (0%)	0 (0%)	1 (5%)	15 (75%)	4 (20%)	3 (1.4%)	89 (41.4 %)	123 (57.2%)
Total (Microscopy)	15 (8.7%)	85 (48.9%)	74 (42.4%)	1 (5%)	15 (75%)	4 (20%)	3 (1.4%)	89 (41.4%)	123 (57.2%)
**PCR results**									
*P. falciparum*	2 (1.2%)	6 (3.4%)	0 (0%)	0 (0%)	3 (15%)	0 (0%)	0 (0%)	0 (0%)	0 (0%)
*P. vivax*	2 (1.2%)	7 (4%)	4 (2.3%)	1 (5%)	0 (0%)	0 (0%)	3 (1.4%)	0 (0%)	0 (0%)
*P. malariae*	0 (0%)	1 (0.6%)	0 (0%)	0 (0%)	0 (0%)	0 (0%)	0 (0%)	0 (0%)	0 (0%)
*P. falciparum + P. vivax*	11 (6.3%)	71 (40.8%)	64 (36.8%)	0 (0%)	12 (60%)	4 (20%)	0 (0%)	88 (41%)	95 (44.2%)
*P. falciparum + P. vivax + P. malariae*	0 (0%)	0 (0%)	6 (3.4%)	0 (0%)	0 (0%)	0 (0%)	0 (0%)	0 (0%)	0 (0%)
Negative	0 (0%)	0 (0%)	0 (0%)	0 (0%)	0 (0%)	0 (0%)	0 (0%)	1 (0.4%)	28 (13%)
Total (PCR)	15 (8.7%)	85 (48.8%)	74 (42.5%)	1 (5%)	15 (75%)	4 (20%)	3 (1.4%)	89 (41.4%)	123 (57.2%)

**Table 3:** Comparison of *Plasmodium* species identified by microscopic and molecular examinations.

Plasmodium Species	Microscopic	Nested PCR	*p*
*P. falciparum*	128 (31.3%)	11 (2.7%)	<0.001
*P. vivax*	34 (8.3%)	16 (3.9%)	< 0.001
*P. malariae*	7 (1.7%)	1 (0.2%)	0.031
*P. falciparum + P. vivax*	5 (1.2%)	346 (84.5%)	<0.001
* P. falciparum + P. vivax + P. malariae	0 (0%)	6 (1.5%)	-
Negative	235 (57.5%)	29 (7.1%)	<0.001

* Computed only for a PxP table.

## Data Availability

The datasets generated and/or analyzed during the current study are not publicly available, as the data are still being used to write further publications and D.A.D.’s dissertation but are available from the corresponding author on reasonable request.
